# Norcantharidin regulates ERα signaling and tamoxifen resistance *via* targeting miR-873/CDK3 in breast cancer cells

**DOI:** 10.1371/journal.pone.0217181

**Published:** 2019-05-23

**Authors:** Xiumei Zhang, Bingfeng Zhang, Panhong Zhang, Lihui Lian, Lianlian Li, Zhihong Qiu, Kai Qian, An Chen, Qiongqing Liu, Yinjie Jiang, Jiajun Cui, Bing Qi

**Affiliations:** 1 The Center for Translational Medicine, Yichun University, Yichun, Jiangxi, P.R. China; 2 College of Chemistry and Bio-engineering, Yichun University, Yichun, Jiangxi, P.R. China; 3 Department of Cell Biology, College of Life Sciences, Shandong First Medical University & Shandong Academy of Medical Sciences, Taian, Shandong, P.R. China; Universitat des Saarlandes, GERMANY

## Abstract

MiR-873/CDK3 has been shown to play a critical role in ERα signaling and tamoxifen resistance. Thus, targeting this pathway may be a potential therapeutic approach for the treatment of ER positive breast cancer especially tamoxifen resistant subtype. Here we report that Norcantharidin (NCTD), currently used clinically as an ani-cancer drug in China, regulates miR-873/CDK3 axis in breast cancer cells. NCTD decreases the transcriptional activity of ERα but not ERβ through the modulation of miR-873/CDK3 axis. We also found that NCTD inhibits cell proliferation and tumor growth and miR-873/CDK3 axis mediates cell proliferation suppression of NCTD. More important, we found that NCTD sensitizes resistant cells to tamoxifen. NCTD inhibits tamoxifen induced the transcriptional activity as well ERα downstream gene expressions in tamoxifen resistant breast cancer cells. In addition, we found that NCTD restores tamoxifen induced recruitments of ERα co-repressors N-CoR and SMRT. Knockdown of miR-873 and overexpression of CDK3 diminish the effect of NCTD on tamoxifen resistance. Our data shows that NCTD regulates ERα signaling and tamoxifen resistance by targeting miR-873/CDK3 axis in breast cancer cells. This study may provide an alternative therapy strategy for tamoxifen resistant breast cancer.

## Introduction

The estrogen receptor (ER), which plays a prominent role in breast cancer, is a member of the nuclear receptor superfamily of ligand-activated transcription factors. There are two different forms of the estrogen receptor, usually referred to as α and β, each encoded by a separate gene (ESR1 and ESR2, respectively) [1,2]. ERα mediates the tumor-promoting effects of estrogens, whereas ERβ inhibits breast cancer cell growth. In common with other nuclear receptors, ER regulates target genes by recruiting transcriptional coregulators and components of the basal transcription machinery [3,4]. The ligand-bound ER, depending on the nature of the ligand, recruits and interacts with coregulatory proteins that can either enhance (coactivators) or repress (corepressors) its transcriptional activity. Ligand-activated ER binds to estrogen-response elements (ERE) of target genes such as TFF1 and c-Myc [3,5].

As approximately 70% of all breast cancers are ERα positive at the time of diagnosis, disruption of ER function is the main therapeutic strategy employed in targeting the disease. The selective estrogen receptor modulator (SERM), tamoxifen, can bind to the ER and block the interaction between estrogen and the ER [6–8]. Through this way, tamoxifen inhibits ER target gene expression and reduces tumor growth. Tamoxifen has been the mainstay of endocrine therapy in both early and advanced breast cancer patients for almost three decades. Unfortunately, up to half of all ER-positive tumors either do not respond to this endocrine therapy or, after initial successful treatment, the tumors recur as endocrine-resistant breast cancer. Thus, tamoxifen resistance presents a major challenge in treating breast cancer [7–9].

MicroRNAs (miRNAs) are 20–22 nucleotide RNAs that negatively regulate gene expression in eukaryotes. Increasingly evidences reveal the key roles of miRNAs in breast cancer initiation and progression. MiRNAs can function as oncogenes or tumor suppressor genes depending on their gene targets. Several recent studies have demonstrated the roles of miRNAs in estrogen signaling and tamoxifen resistance [10–16]. Our group recently reported that miR-873 regulates estrogen receptor signaling and tamoxifen resistance. MiR-873 is down-regulated in breast cancer and its overexpression sensitized breast cancer cells to tamoxifen in vitro and in vivo. CDK3 is the direct target of miR-873 and is overexpressed in breast cancer. Mir-873 exerted its functions through inhibiting CDK3 expression in breast cancer. Thus, targeting miR-873/CDK3 may be a potential therapeutic approach for the treatment of ER positive breast cancer especially tamoxifen resistant breast cancer [17].

Here, we, we found that Norcantharidin (NCTD) significantly increases miR-873 expression. NCTD inhibits breast cancer cell growth *in vitro* and *in vivo* through regulating miR-873/CDK3 axis. More important, NCTD sensitized resistant cancer cells to tamoxifen.

## Results

### Norcantharidin (NCTD) regulates miR-873/CDK3expressions in breast cancer cells

**Fig 1:**
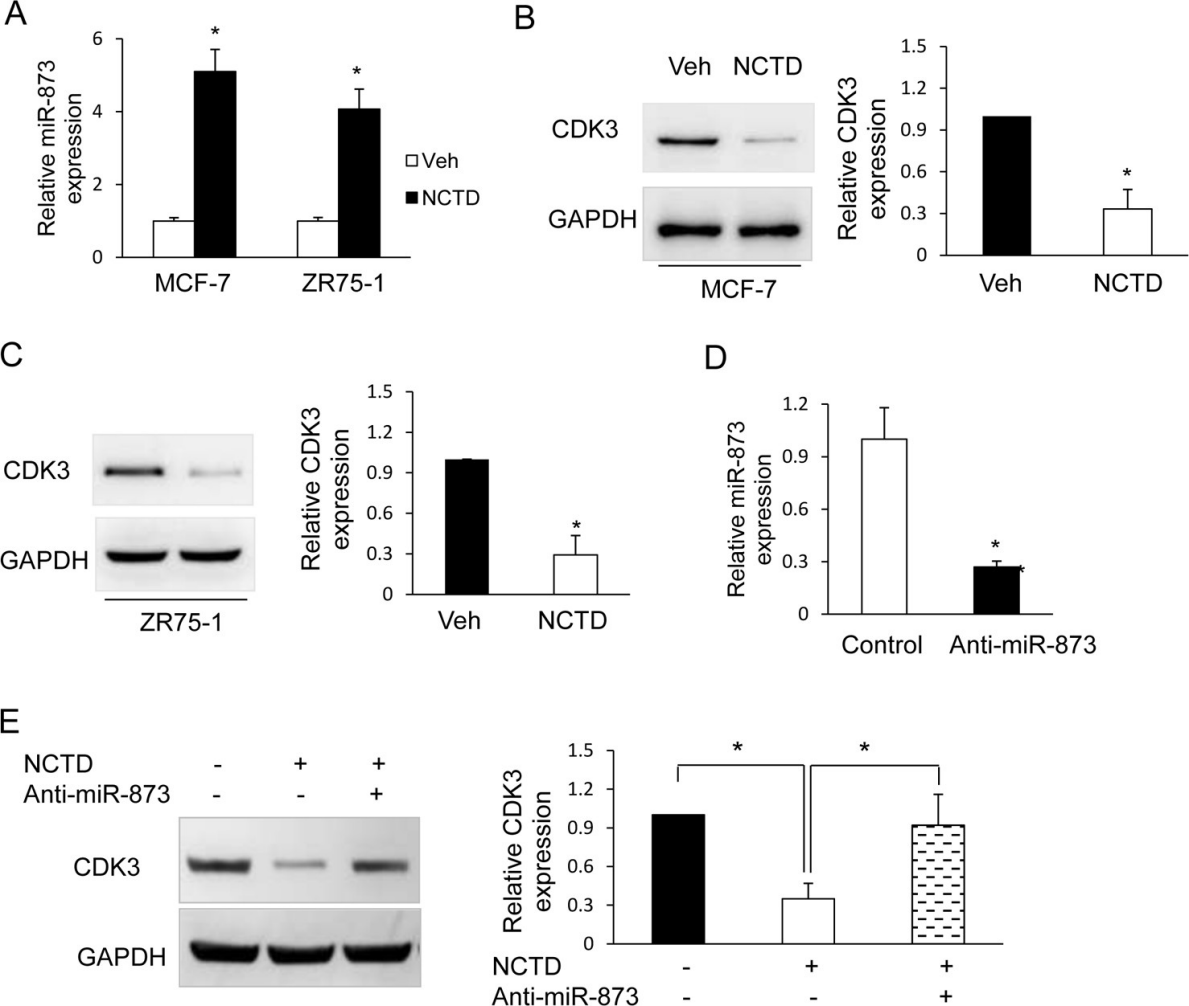
NCTD regulates miR-873/CDK3 axis. **(A)** Real-time PCR analysis of miR-873 level in MCF-7 and ZR75-1 cells treated with NCTD. MCF-7 and ZR75-1 cells were treated with vehicle (Veh) or 25μM NCTD for 24h and then cells were harvested to perform real-time PCR. **(B) and (C)** MCF-7 and ZR75-1 cells were treated with 25μM NCTD. 24h later cells were harvested to perform western blot using anti-CDK3 antibody. Quantifications of western blot are shown in the right column. **(D)** Real-time PCR analysis of miR-873 level in MCF-7 cells transfected with anti-miR-873 or control oligo. **(E)** MCF-7 cells were transfected with anti-miR-873 or control oligo and then treated with Vehicle (Veh) or 25μM NCTD for 24h. Western blot assays were performed to detect the expression CDK3. Data are expressed as mean ± SD. * P < 0.05.

Our previous study shows that miR-873/CDK3 axis plays a critical role in ERα signaling and tamoxifen resistance. Targeting this pathway may be a potential therapeutic approach for the treatment of ER positive breast cancer especially tamoxifen resistant subtype [17]. Since natural compounds have been an important source of many clinically useful anti-cancer agents, here we tried to screen naturally derived compounds regulating miR-873 expression using real-time PCR. As a result, we found that NCTD increased significantly miR-873 expression in MCF-7 and ZR75-1 cells (Fig 1A).

CDK3 is the target of miR-873 to regulate ERα signaling and tamoxifen resistance. Then, we investigated the effect of NCTD on CDK3 expression and Western blot assays showed that NCTD inhibited CDK3 expression (Fig 1B and 1C). To determine whether NCTD inhibits CDK3 expression *via* miR-873, we used anti-miR-873 inhibitor to diminish miR-873 expression in MCF-7 cells. As expected, the anti-miR-873 inhibitor oligo effectively inhibited miR-873 expression, whereas the control oligo had no effect (Fig 1D). Importantly, suppression of the normal expression of miR-873 in MCF-7 cells significantly diminished the inhibitory effect of NCTD on CDK3 expression (Fig 1E).

### NCTD regulates ERα signaling in breast cancer cells

**Fig 2:**
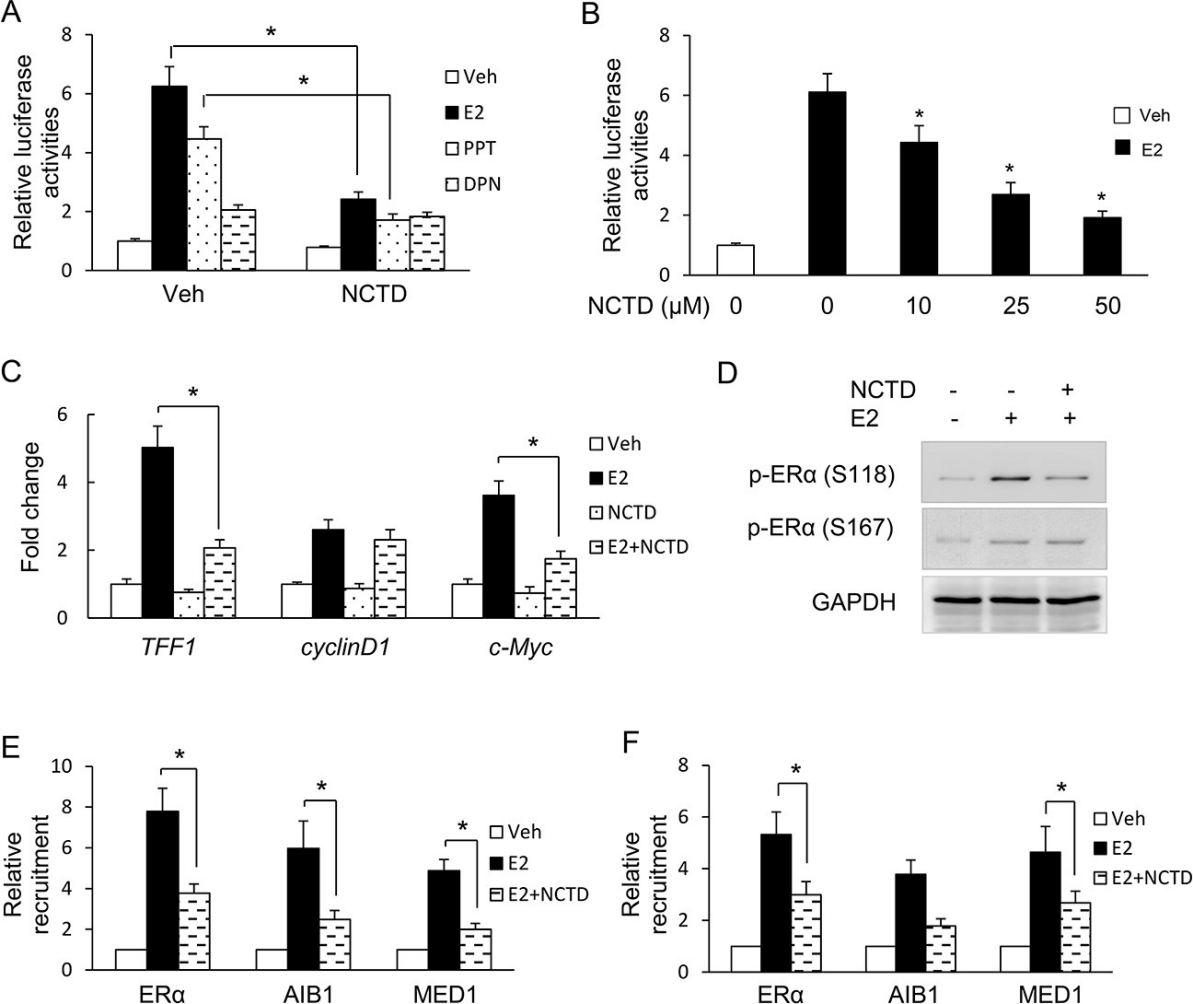
NCTD inhibits ERα transcriptional activity in breast cancer cells. **(A)** NCTD inhibited ERE (estrogen response element) reporter gene activities. MCF-7 cells were transfected with plasmids expressing ERE-TK-LUC reporter and pRL-TK (internal control) and followed by vehicle, E2, PPT, DPN or NCTD treatment as indicated for 24 hours. The relative luciferase values are expressed as mean ± S.E. **(B)** NCTD inhibited ER transcriptional activities in a dose-dependent manner. Cells indicated above were treated with E2 and different concentration of NCTD as indicatd and the relative luciferase activities were detected. **(C)** MCF-7 cells were treated with E2 or and 25μM NCTD for 24h. Real-time PCR assays were performed to detect the effect of NCTD on ERα downstream gene expressions as indicated. **(D)** MCF-7 cells were treated with E2 or and 25μM NCTD for 24h. Western blot assays were performed to detect the effect of NCTD on ERα phosphorylation level as indicated. **(E, F)** NCTD inhibited the recruitments of ERα and its coregulators. MCF-7 were treated with 25μM NCTD and followed by ChIP to detect the recruitments of ERα and its coregulators on the promoter of *TFF1* **(E)** and *c-Myc* **(F)**. Data are expressed as mean ± SD. * P < 0.05.

To investigate the role of NCTD in ER transcriptional activities, the ERE-Luc was transfected into breast cancer cells and then cells were treated with NCTD. As shown in Fig 2A and 2B, NCTD inhibited luciferase reporter activities in presence of E2 in MCF-7 cells. Interestingly, NCTD significantly decreased reporter gene activity in response to the ERα-specific agonist propylpyrazoletriol (PPT) but not to the ERβ-specific agonist, diarylpropionitrile (DPN). These results indicate that NCTD inhibits ERα but not ERβ transcriptional activity. We also found NCTD inhibited ER transcriptional activities in T47D cells (S1 Fig)

Then, we also examined the effect of NCTD on several well-known endogenous ERα target genes. NCTD treatment inhibited TFF1 and c-Myc expression at the mRNA level (Fig 2C). ERα function is regulated by phosphorylation by various protein kinases [18]. Thus, we examined the effect of NCTD on ERα phosphorylation. In the presence of E2, NCTD inhibited phosphorylation of S118 but not S167 (Fig 2D). Finally, we performed ChIP to investigate the effect of NCTD on the occupancies of ERα and its co-activators. As a result, NCTD inhibited the recruitments of ERα and its co-activators (MED1 and AIB1) to *TFF1*and *c-Myc* promoter (Fig 2E and 2F).

### NCTD regulates ERα signaling via miR-873/CDK3

**Fig 3:**
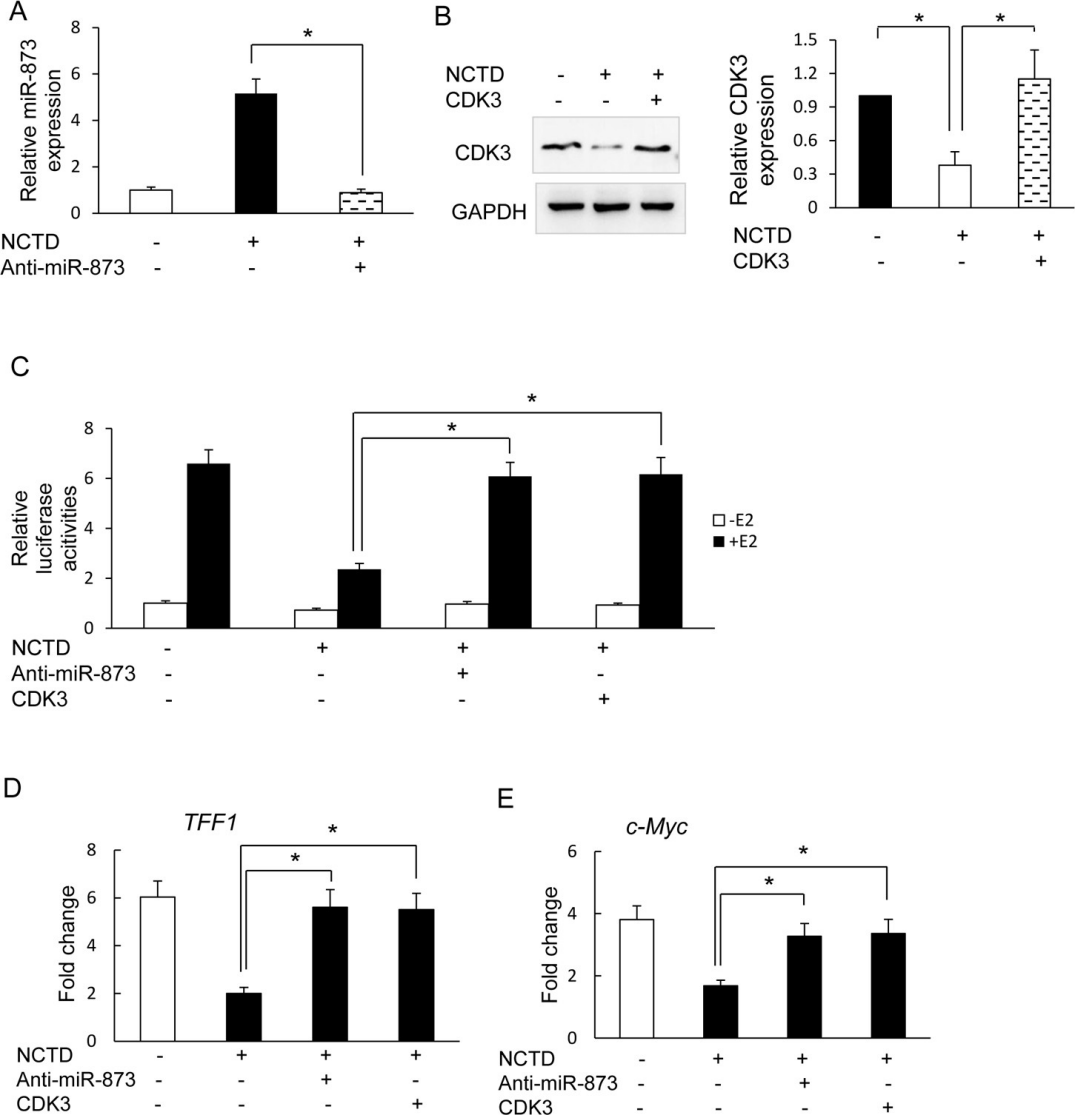
NCTD inhibits ER transcriptional activity and cell growth *via* miR-873/CDK3 axis. **(A)** Real-time PCR analyses of miR-873 expression in MCF-7 cells which were transfected with anti-miR-873 oligo in the absence and the presence of 25μM NCTD. **(B)** Western blot analyses of CDK3 protein levels after infections with lentivirus expressing CDK3 into MCF-7 cells in the absence and the presence of 25μM NCTD. Quantifications of western blot are shown in the right column. **(C)** MCF-7 cells which transfected with anti-miR-873 oligo or CDK3 expression vector were co-transfected with plasmids expressing ERE-TK-LUC reporter and pRL-TK (internal control) and followed by vehicle, 25μM NCTD or E2 treatment for 24 hours. The relative luciferase values are expressed as mean ± S.E. **(D, E)** MCF-7 cells which transfected with anti-miR-873 oligo or CDK3 expression vector were treated with vehicle, 25μM NCTD or 100nM E2 treatment for 24 hours. Real-time PCR assays were then performed to measure *TFF1* and *c-Myc* expression. Data are expressed as mean ± SD. * P < 0.05.

To further explore whether NCTD inhibits ERα transcriptional activities *via* miR-873/CDK3, we re-introduced the anti-miR-873 inhibitor or CDK3 coding sequence without the 3’UTR into MCF-7 cells. Real-time PCR and western blots showed that miR-873 and CDK3 expression restored to similar levels in NCTD treated cells as untreated cells (Fig 3A and 3B). We then measured ERα transcriptional activity and found that knockdown of miR-873 and re-expression of CDK3 both diminished the inhibitory effect of NCTD on ERα transcriptional activities in MCF-7 cells (Fig 3C). Real-time PCR assays showed that knockdown of miR-873 and re-expression of CDK3 could also restore ER target genes such as TIFF1 and c-Myc expression which were inhibited by NCTD (Fig 3D and 3E).

### NCTD inhibits cell growth in vitro and in vivo via targeting miR-873/CDK3

**Fig 4:**
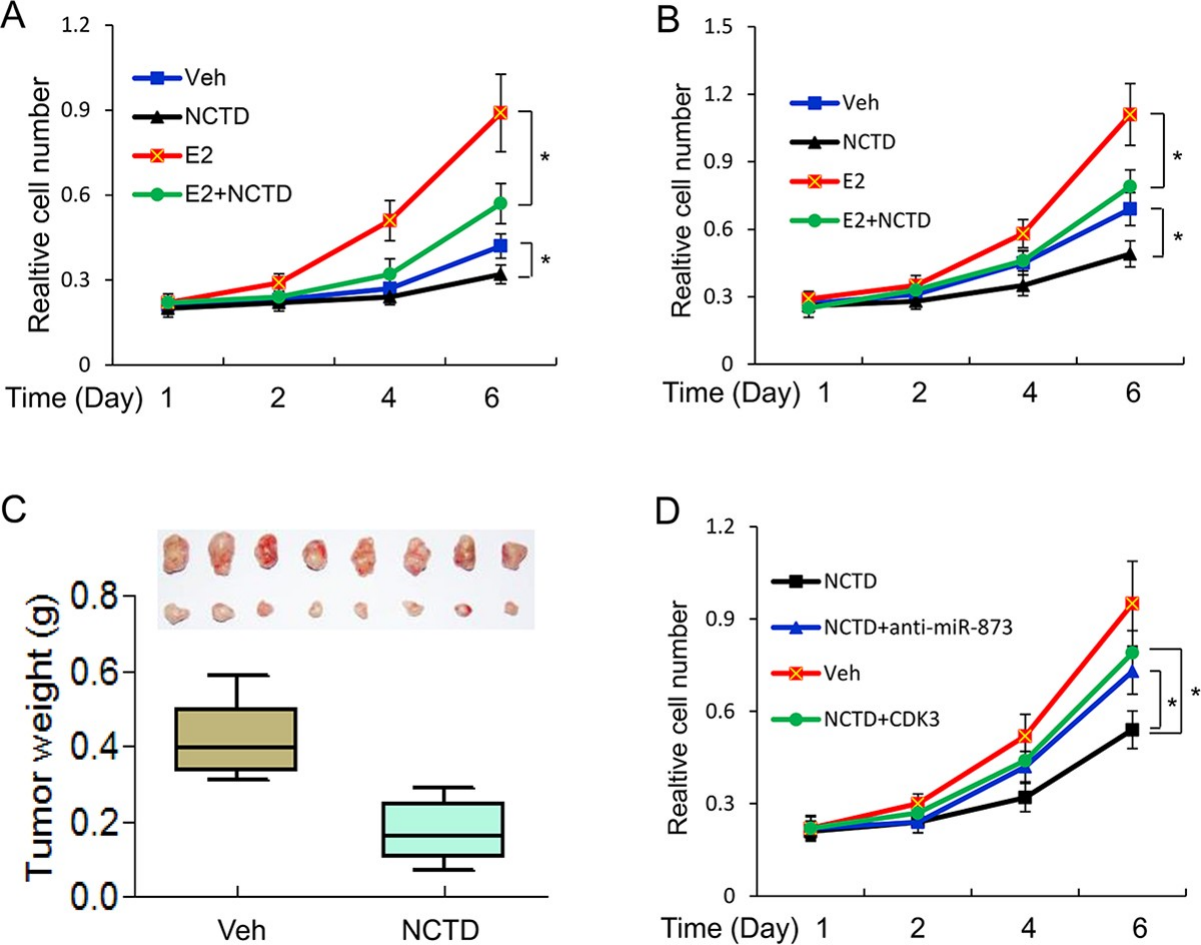
NCTD inhibits cell growth *in vitro* and *in vivo*. **(A, B)** MTT assays of cell proliferation in MCF-7 **(A)** and ZR75-1 **(B)** cells in the presence of 25μM NCTD. **(C)** NCTD inhibited tumor growth in a human breast cancer MCF-7 xenograft mouse model. Tumor weights were calculated and shown as a box-plot with median and whiskers from minimum to maximum. **(D)** NCTD inhibited cell growth *via* miR-873/CDK3 axis. MCF-7 cells were transfected with anti-miR-873 oligo or CDK3 expression vector and then subjected to 25μM NCTD and 100nM E2 treatment for 6 days. MTT assay was performed to detect the cell proliferation. Data are expressed as mean ± SD. * P < 0.05.

Our above data indicated a key role for NCTD in ERα-mediated transcription. As well known, ERα determines the growth of ER-positive breast cancer cells. Thus, we further assessed the role of NCTD in the growth of this type of breast cancer cells. We first performed cell proliferation assays in MCF-7 and ZR75-1. As expected, estrogen treatment strongly promoted the proliferation of MCF-7 and ZR75-1 cells. NCTD significantly diminished cell proliferation of MCF-7 and ZR75-1 cells (Fig 4A and 4B). Moreover, we found that NCTD suppressed MCF-7 cell proliferation in a dose dependent manner (S2 Fig). Next, we determined the role of NCTD in tumor growth of nude mice. We found that NCTD significantly reduced tumor growth *in vivo* (Fig 4C).

We further investigated whether NCTD inhibits cell proliferation *via* targeting miR-873/CDK3. As a result, we found that knockdown of miR-873 and re-expression of CDK3 both diminished the inhibitory effect of NCTD on cell proliferation in MCF-7 cells (Fig 4D).

### NCTD regulates tamoxifen resistance in breast cancer cells

**Fig 5:**
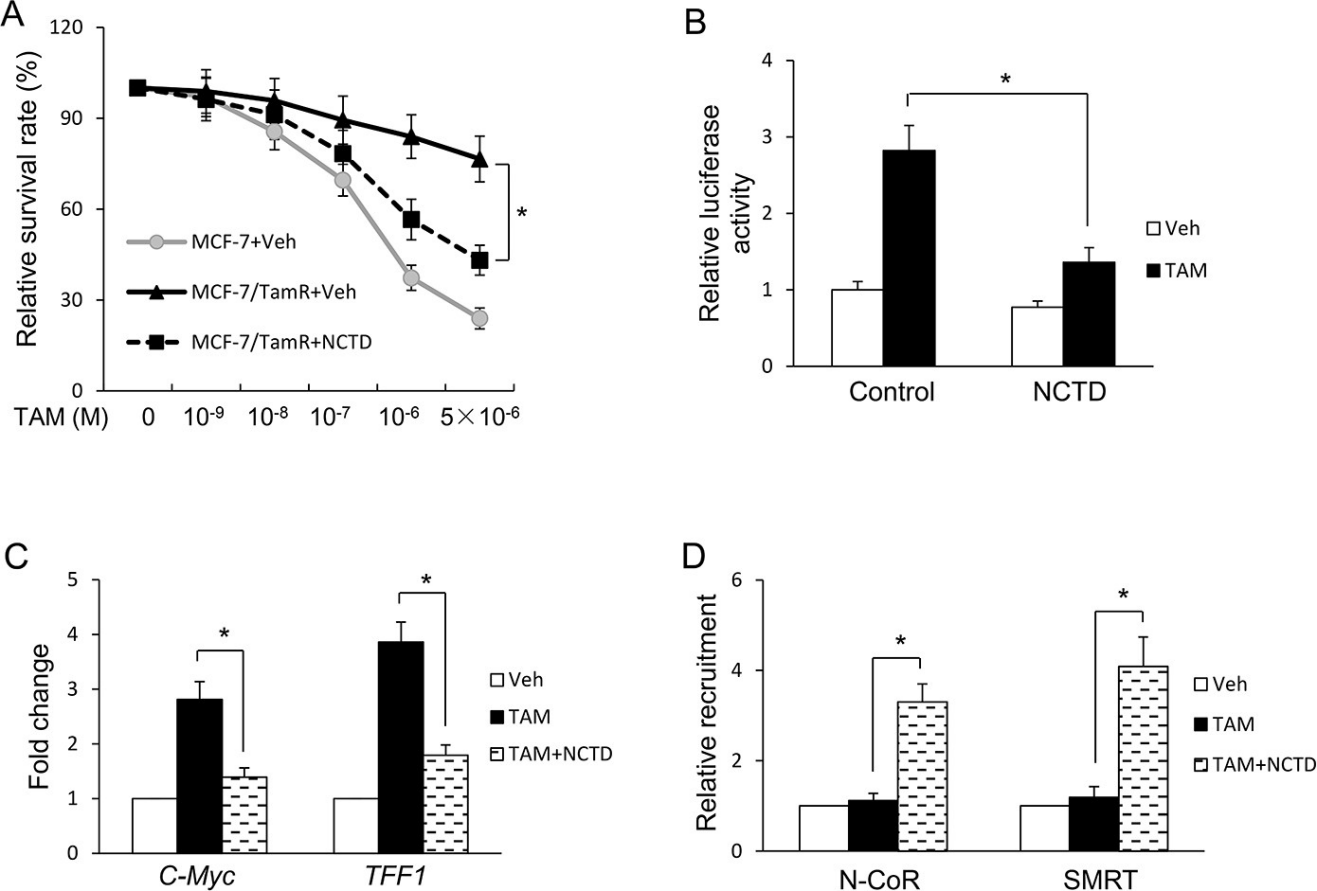
NCTD sensitizes resistant cells to tamoxifen treatment. **(A)** MCF-7/TamR cells were treated with indicated amount of 4-hydroxytamoxifen (TAM) and/or 25μM NCTD for 7 days. Cells were then harvested and assessed for cell proliferation by MTT assays. **(B)** MCF-7/TamR cells were along transfected with plasmids expressing ERE-TK-LUC reporter and PRL-TK (internal control) and followed by vehicle, TAM and/or NCTD treatment for 24 hours. The relative luciferase values are expressed as mean ± S.E. **(C)** MCF-7/TamR cells were treated with treated with vehicle (Veh), TAM and/or NCTD for 24 h and *TFF1* and *c-Myc* gene expression was detected by Real-time PCR. **(D)** MCF-7/TamR cells were treated with treated with NCTD for 24h and then followed by TAM treatment for 1 h. ChIP assays were performed to detect the recruitments of N-CoR and SMRT on the promoter of *TFF1*. Data are expressed as mean ± SD. * P < 0.05.

Our previous study showed that miR-873/CDK3 axis plays a critical role in tamoxifen resistance. We decided to carry out experiments to investigate whether NCTD could regulate tamoxifen resistance in breast cancer cells. For this purpose, MCF-7/TamR cells were treated with tamoxifen and/or NCTD and then MTT assays were performed. We found that NCTD treatment was capable of sensitizing MCF-7/TamR cells to tamoxifen (Fig 5A).

Our and other studies have shown that tamoxifen plays an agonist role on ERα-mediated transcription in tamoxifen resistant cells. To determine the effect of NCTD on ERα transcriptional activity and estrogen-responsive gene expression in response to tamoxifen, we decided to carry out a series of experiments. Reporter gene activity assays showed NCTD could inhibit ERα transcriptional activity induced by tamoxifen in MCF-7/TamR cells (Fig 5B). We then examined the effects of NCTD on tamoxifen-induced expression of endogenous ERα target genes in MCF-7/TamR cells. As shown in Fig 5C, our results indicate that NCTD inhibited tamoxifen induced expression of ERα target genes such as TFF1 and c-Myc.

N-CoR and SMRT were shown to play important roles in the anti-proliferative action of tamoxifen [17,19]. Constant with our previous study, tamoxifen was not able to recruit N-CoR and SMRT onto the promoters of ERα target genes in MCF-7/TamR cells. Importantly, NCTD treatment effectively restored the recruitments of N-CoR and SMRT recruitment by tamoxifen to *TFF1* promoter (Fig 5D) and *c-Myc* promoter (S3 Fig).

### NCTD regulates tamoxifen resistance via miR-873/CDK3 in breast cancer cells

**Fig 6:**
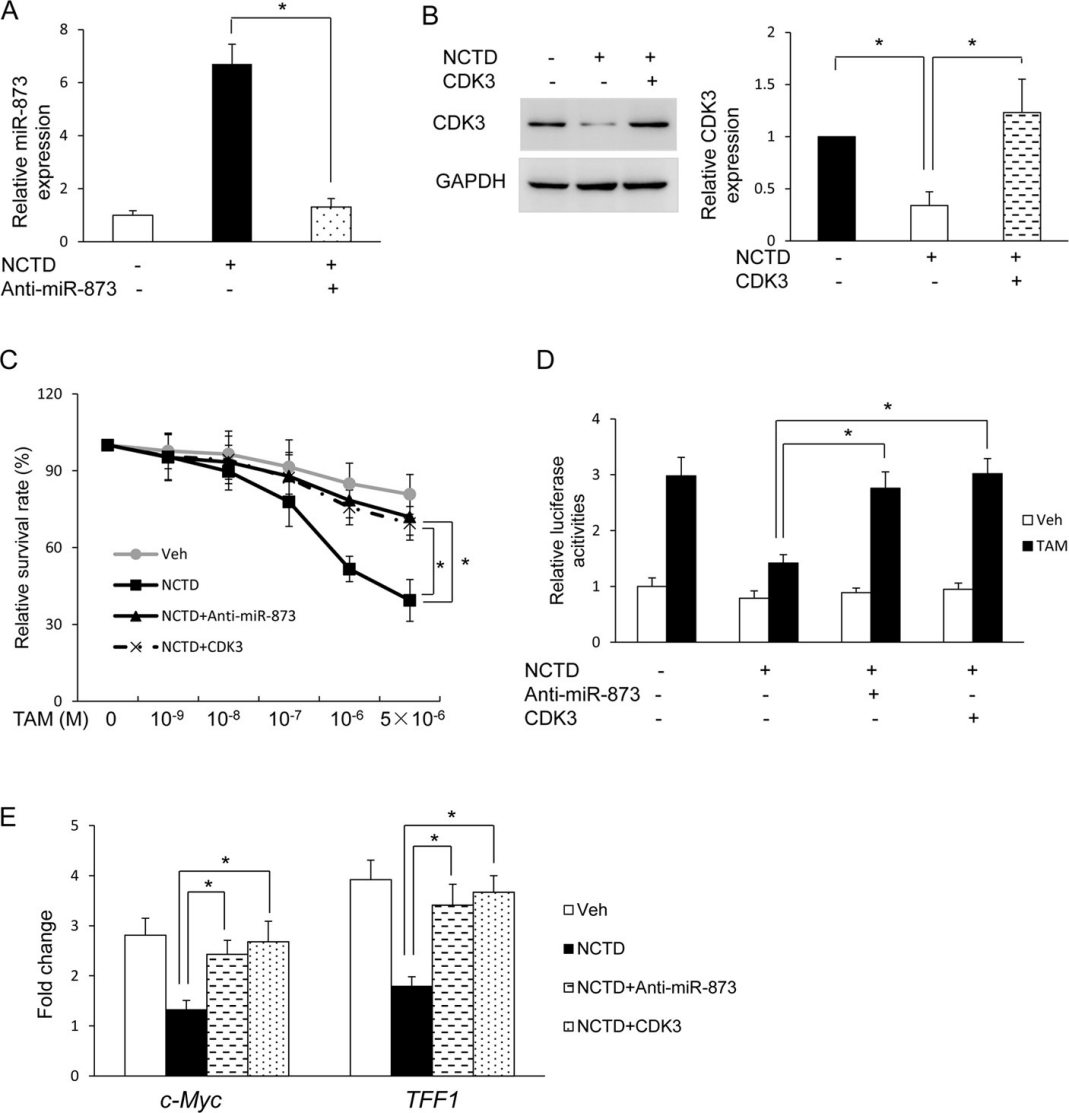
NCTD regulates tamoxifen resistance *via* miR-873/CDK3 axis. **(A)** Real-time PCR analyses of miR-873 expression in MCF-7/TamR cells which were transfected with anti-miR-873 oligo in the absence and the presence of 25μM NCTD. **(B)** Western blot analyses of CDK3 protein levels after infections with lentivirus expressing CDK3 into MCF-7/TamR cells in the absence and the presence of 25μM NCTD. Quantifications of western blot are shown in the right column. **(C)** MCF-7/TamR cells which transfected with anti-miR-873 oligo or CDK3 expression vector and followed by vehicle, 25μM NCTD or TAM treatment for 7 days. MTT assays were then performed to determine the tamoxifen response. **(D)** MCF-7/TamR cells which transfected with anti-miR-873 oligo or CDK3 expression vector were co-transfected with plasmids expressing ERE-TK-LUC reporter and pRL-TK (internal control) and followed by vehicle, 25μM NCTD or TAM treatment for 24 hours. The relative luciferase values are expressed as mean ± S.E. Cells mentioned in (D) were harvested for real-time PCR assays to detect the expression of TFF1 and c-Myc **(E).** Data are expressed as mean ± SD. * P < 0.05.

To further explore whether NCTD regulates tamoxifen resistance *via* miR-873/CDK3, we re-introduced the anti-miR-873 inhibitor or CDK3 coding sequence without the 3’UTR into MCF-7/TamR cells (Fig 6A and 6B). MTT assays revealed that knockdown of miR-873 and re-expression of CDK3 both reversed the effect of NCTD on tamoxifen resistance in MCF-7/TamR cells (Fig 6C). We also found that knockdown of miR-873 and re-expression of CDK3 could diminish the effect of NCTD on tamoxifen induced ERα transcriptional activity and estrogen-responsive gene expression in MCF-7/TamR cells (Fig 6D and 6E). Taken together, our results show that NCTD sensitizes resistant cells to tamoxifen *via* targeting miR-873/CDK3.

## Discussion

In this study, we have demonstrated the role of NCTD in regulating ER signaling and tamoxifen resistance of human breast cancer cells *via* miR-83/CDK3 axis. We found that: (i) NCTD increases miR-873 expression and inhibits CDK3 expression in MCF-7 cells; (ii) NCTD inhibits ER signaling and breast cell growth *via* targeting miR-873/CDK3 in vitro and in vivo; (iii) NCTD treatment sensitizes resistant cells to tamoxifen though miR-873/CDK3 axis; (iv) NCTD is able to restore the tamoxifen-induced recruitment of N-CoR and SMRT to the promoters of ER target genes. Taken together, these findings support a key role for NCTD in regulating ER signaling and tamoxifen resistance in human breast cancer.

Studies have revealed that miRNAs are frequently aberrantly expressed in cancer. MiRNAs have been implicated in almost all aspects of cancer biology, including proliferation, apoptosis, invasion, metastasis, angiogenesis and drug resistance [15,16,20–22]. Our group has identified that an unknown function miRNA, miR-873 is downregulated in breast cancer. MiR-873 inhibits the transcriptional activity of ERα but not ERβ there by modulating ERα phosphorylation. MiR-873 was also found to inhibit the proliferation of breast cancer cells and tumor growth. We then noted that miR-873 reverses tamoxifen resistance by targeting CDK3. These data suggest the role of miR-873/CDK3 in regulating ER signaling and tamoxifen resistance in breast cancer cells [17]. Despite the fact that the majority of breast cancer patients have estrogen receptor (ER) α-positive tumors, most of the patients are or soon develop resistance to endocrine therapy. Until now, tamoxifen resistance remains a major challenge in breast cancer treatment [6,9,19,23,24]. Our data provides miR-873/CDK3 axis is a potential target to treat ER positive cancer especially tamoxifen resistant subtype, but how to target this axis is a challenge. For some serious problems such as delivery, microRNA drug has been not used in clinic. As for CDK3, there is no specific inhibitor was available although CDK4/6 inhibitors have been approved by FDA. Naturally derived compounds become key role players in future cancer treatments. More than 30% of anti-cancer agents have their origin in natural sources [25,26]. Thus, to screen natural compounds of targeting miR-873/CDK3 is a promising strategy. In this study, we employed a real-time PCR based screen for natural compounds to target miR-873. As a result, we found several compounds could regulate miR-873 expression, of which norcantharidin (NCTD) significantly increases miR-873 expression.

NCTD is the demethylated analog of cantharidin isolated from blister beetles (*Mylabris phalerata* Pall.). NCTD is synthesized to reduce the toxic side effects and retain the bioactivity of cantharidin. It is now used clinically to treat liver cancer in china. It has been reported that NCTD could inhibits cell proliferation, invasion, metastasis and angiogenesis in various types of cancer cells [27–29]. Unlike the conventional chemotherapeutics, NCTD is preferentially toxic to cancer cells than normal cells, making this small molecule attractive in cancer treatment. Recent studies showed that NCTD could overcome doxorubincin resistance in breast cancer cells and Hepatocyte growth factor (HGF) induced resistance to epidermal growth factor receptor tyrosine kinase inhibitors (EGFR-TKI) in lung cancer cells. These studies broaden the usage of NCTD in cancer treatment [28,30,31]. Here, we found that NCTD possesses the anti-cancer properties in ER positive cancer cells and tamoxifen resistant cells. This suggests that NCTD might be used to treat not only ER positive cancer but also tamoxifen resistant cancer. Since NCTD is already used clinically for cancer treatment, it is promising for combination NCTD with tamoxifen to treat ER positive and even tamoxifen resistant breast cancer.

Recent studies showed that CD44^+^CD24^-^ cell population (breast cancer stem cell subpopulation) in MCF-7 cells were resistant to tamoxifen treatment, however, tamoxifen resistant cells harbored higher number of CD44^+^CD24^-^ cell population than MCF-7 cells [32,33]. This crosstalk between cancer stem cells and tamoxifen resistant cells suggests that cancer stem cells play a vital role in tamoxifen resistance acquirement. NCTD has been shown to repress cancer stem cell expansion by inhibiting ß-catenin in pancreatic cancer [29,34]. Thus, it is very interesting to study the molecular mechanisms for NCTD affect this crosstalk.

Compared with other members of the CDK family, little is known about CDK3. While CDK3 is closely related to CDK2, it is not required for cell cycle progression in normal cells. CDK3 exhibits low expression in human tissue and spontaneous mutational inactivation in the germline of laboratory mice. In breast cancer and glioblastoma tissue, CDK3 is overexpressed [35–38]. CDK2 inhibitor can selectively target CD44^+^CD24^-^ subpopulation and restores chemo-sensitivity in breast cancer [39]. During the preparation of this manuscript, miR-873/CDK3 has been shown to function in cancer stemness of lung cancer cells [40]. These reports imply that miR-873/CDK3 mediates the potential effect of NCTD on the crosstalk between tamoxifen resistance and cancer stemness in breast cancer cells. Further experiments on the cell and mouse models are underway.

Taken together, our data shows that NCTD overcomes tamoxifen resistance by targeting miR-873/CDK3 axis in breast cancer cells. This study may identify a novel therapeutic target of NCTD for breast cancer treatment. Combination NCTD with tamoxifen would be a promising strategy to treat ER positive and even tamoxifen resistant breast cancer.

## Materials and methods

### Cell culture

The human breast cancer cell lines MCF-7, ZR75-1, T47D and tamoxifen resistant cells MCF-7/TamR were described previously [17]. Cell lines were authenticated on the basis of viability, recovery, growth, and morphology. The expression status of ER was further confirmed by Western blot before they were used in experiments. All cells were cultured in Dulbecco's Modified Eagle's Media (DMEM) medium containing 10% FBS (Hyclone, Thermo fisher scientific, Florence, KY, USA) at 37°C with 5% CO2 in tissue culture incubators. Norcantharidin (NCTD), 17β-estradiol (E2), propylpyrazoletriol (PPT), diarylpropionitrile (DPN) and 4-hydroxytamoxifen (TAM) were purchased from Sigma (St Louis, MO, USA). The concentrations of these drugs used in this study were according to previous studies [17, 41].

### Plasmids and lentiviral vector preparation

The plasmid pERE-TK-Luc, pRL-TK, lentiviral construct pCDH-CMV-CDK3 and lentivirus package was described previously [17,19]. Anti-miR-873 inhibitor and control oligonucleotides were purchased from Invitrogen (Invitrogen, Carlsbad, CA, USA).

### Transient transfections and reporter gene assays

For transfection, cells were plated in 24-well plates containing phenol red-free RPMI 1640 medium supplemented with 10% charcoal-stripped FBS, and the plasmids were transfected with Lipofectamine2000 (Invitrogen, Carlsbad, CA, USA). Cells were treated with NCTD, E2 or TAM for 24 hours and then harvested for the dual luciferase assay. The dual luciferase reporter assay system (Promega, Madison, WI, USA) was employed to measure the luciferase activity.

### Real-time PCR

Total RNA was isolated from cells with an Rneasy Mini kit (Qiagen, Hilden, Germany) or Trizol (Invitrogen, Carlsbad, CA, USA) reagent according to the manufacturer’s instructions. Total RNA from each sample was reverse transcribed using SuperScript III Reverse Transcriptase (Invitrogen) followed by Real-time PCR. Primers for miR-873 were purchased from (Exiqon, Vedbaek, Denmark). Primer sequences for TFF1 and c-Myc were described previously [17]. Real-time PCR was performed with SYBR Green PCR Master Mix reagents using an ABI Prism 7700 Sequence Detection System (Applied Biosystems, Foster City, CA, USA).

### Western blot

Cells were lysed and total proteins separated using 10% SDS-PAGE gel and then transferred to PVDF membrane. The membrane was blocked using BSA for 1 h at room temperature (RT). After incubation with primary antibody against CDK3 antibody (Abcam, Cambridge, MA, USA) for 2h at RT, the membrane was washed extensively and then incubated with secondary horseradish peroxidase–conjugated antibody for 1h at RT. Blots were developed with the ECL plus western blotting detection system (Thermo Fischer Scientific, Waltham, MA USA).

### Chromatin immunoprecipitation (ChIP)

Cells were treated with NCTD for 24h. Before harvesting, cells were treated for 1h with vehicle (ethanol), 100nM E2 or 1μM TAM and immediately fixed by adding 37% formaldehyde to the medium to a final concentration of 1%. After PBS washing, cells were harvested and lysised. The nuclear lysates were sonicated to generate an average DNA size of 0.5–1 kb, and then immunoprecipitation was performed with anti-ERα, anti-N-CoR or anti-SMRT (Santa Cruz, CA, USA). The recruitments of proteins on the TFF1 and c-Myc promoter were detected using real-time PCR. The primers for TFF1 and c-Myc promoter were described previously [17, 19].

### Cell proliferation assays

MTT assay was described previously [17, 42]. Cells were treated with NCTD, vehicle, E2 or TAM. The proliferation was determined using a CellTiter 96 nonradioactive cell proliferation assay kit (Promega, Madison, MI, USA) according to the manufacturer’s instructions.

### Animal experiment

All the experimental procedures involving animals were conducted in accordance with the National Institutes of Health guide for the care and use of laboratory animals (NIH publications No.8023, revised 1978). The protocol has been approved ethically by the Administration Committee of Experimental Animals, Medicine School, Yichun University, Yichun, China and the protocol number is IACUC-2016012. Two days after implantation of estrogen pellets (E2, 0.36 mg/pellet, 60-day release) (Innovative Research of America, Sarasota, FL, USA), 1 × 10^7^ tumor cells were injected into the abdominal mammary fat pad of 6-week-old female nude mice. When tumors reached the volume of approximately 100 mm^3^, we randomly allocated the mice to two groups and 8 mice in each group. Mice were i.p. injected with 30 mg / kg NCTD twice each week for 6 weeks. Excised tumors were weighed, and portions were frozen in liquid nitrogen.

### Statistical analysis

All the experiments were repeated at least three times. Presented data are the mean ± SEM of three experiments. Student’s *t*-test for multiple group comparisons was performed using SPSS. *P* < 0.05 was considered statistically significant.

## Supporting information

S1 Fig NCTD decreased ERα transcriptional activities in T47D cells. T47D cells were transfected with plasmids expressing ERE-TK-LUC reporter and pRL-TK (internal control) and followed by vehicle, E2, or/and NCTD treatment as indicated for 24 hours. The relative luciferase values are expressed as mean ± S.E.
(TIF)

S2 Fig NCTD inhibits MCF-7 cell proliferation in a dose dependent manner. MCF-7 cells were treated with various concentrations of NCTD as indicated and E2 for 6 days. Cells were subjected to MTT assay. Data are expressed as mean ± SD. Ns, not significant, * P < 0.05, ** P < 0.05.
(TIF)

S3 Fig NCTD restores the recruitments of N-CoR and SMRT by tamoxifen onto *c-MYC* promoter in MCF-7/TamR cells. MCF-7/TamR cells were treated with treated with NCTD for 24h and then followed by TAM treatment for 1 h. ChIP assays were performed to detect the recruitments of N-CoR and SMRT on the promoter of *TFF1*. Data are expressed as mean ± SD. * P < 0.05.
(TIF)
