# Supplementary figures and images for: Norcantharidin regulates ERα signaling and tamoxifen resistance via targeting miR-873/CDK3 in breast cancer cells
Source: PLoS One. 2019 May 23;14(5):e0217181. doi: 10.1371/journal.pone.0217181 (PMC6532885; doi:10.1371/journal.pone.0217181)

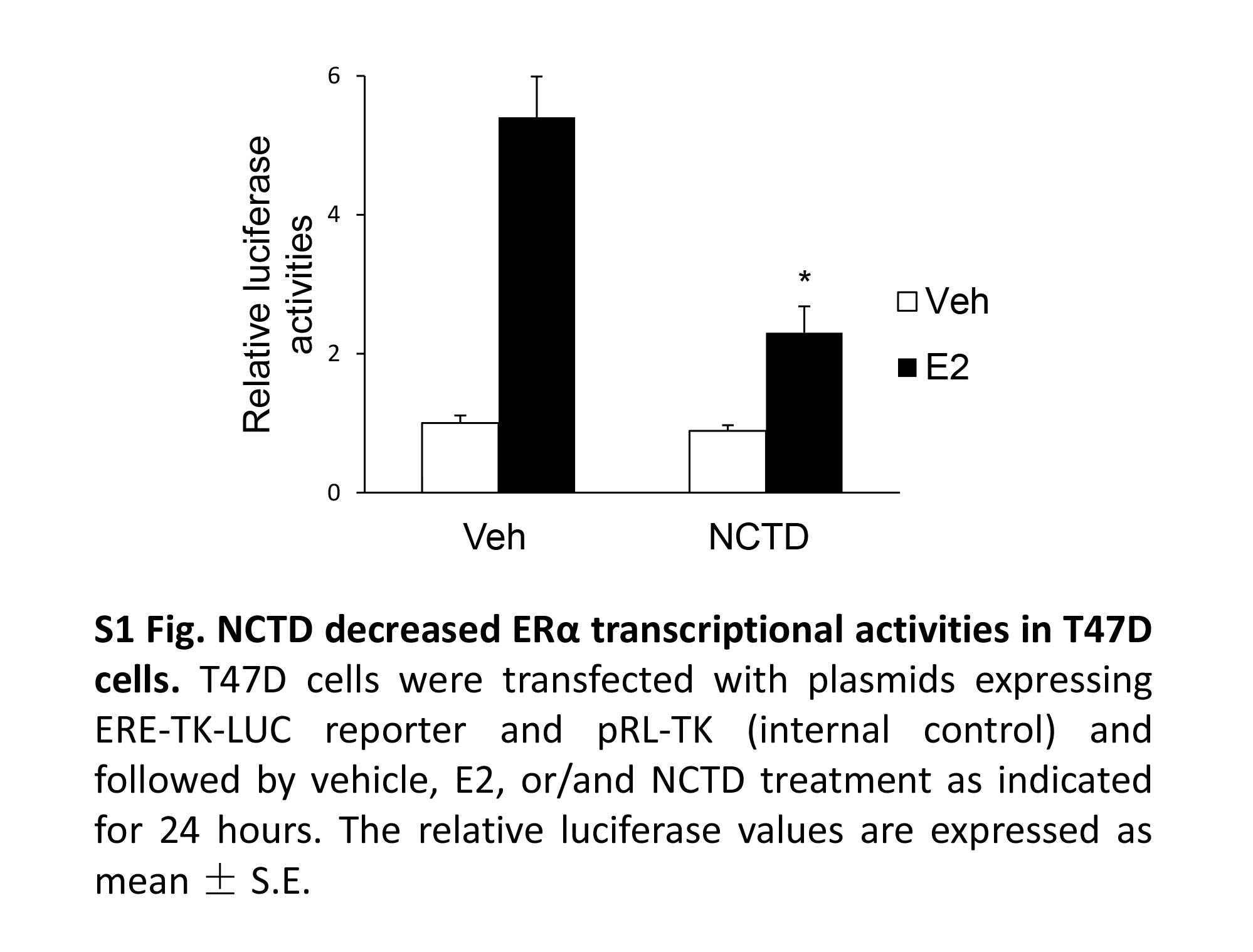

Supplement: S1 Fig — T47D cells were transfected with plasmids expressing ERE-TK-LUC reporter and pRL-TK (internal control) and followed by vehicle, E2, or/and NCTD treatment as indicated for 24 hours. The relative luciferase values are expressed as mean ± S.E. (TIF) [file pone.0217181.s001.tif]

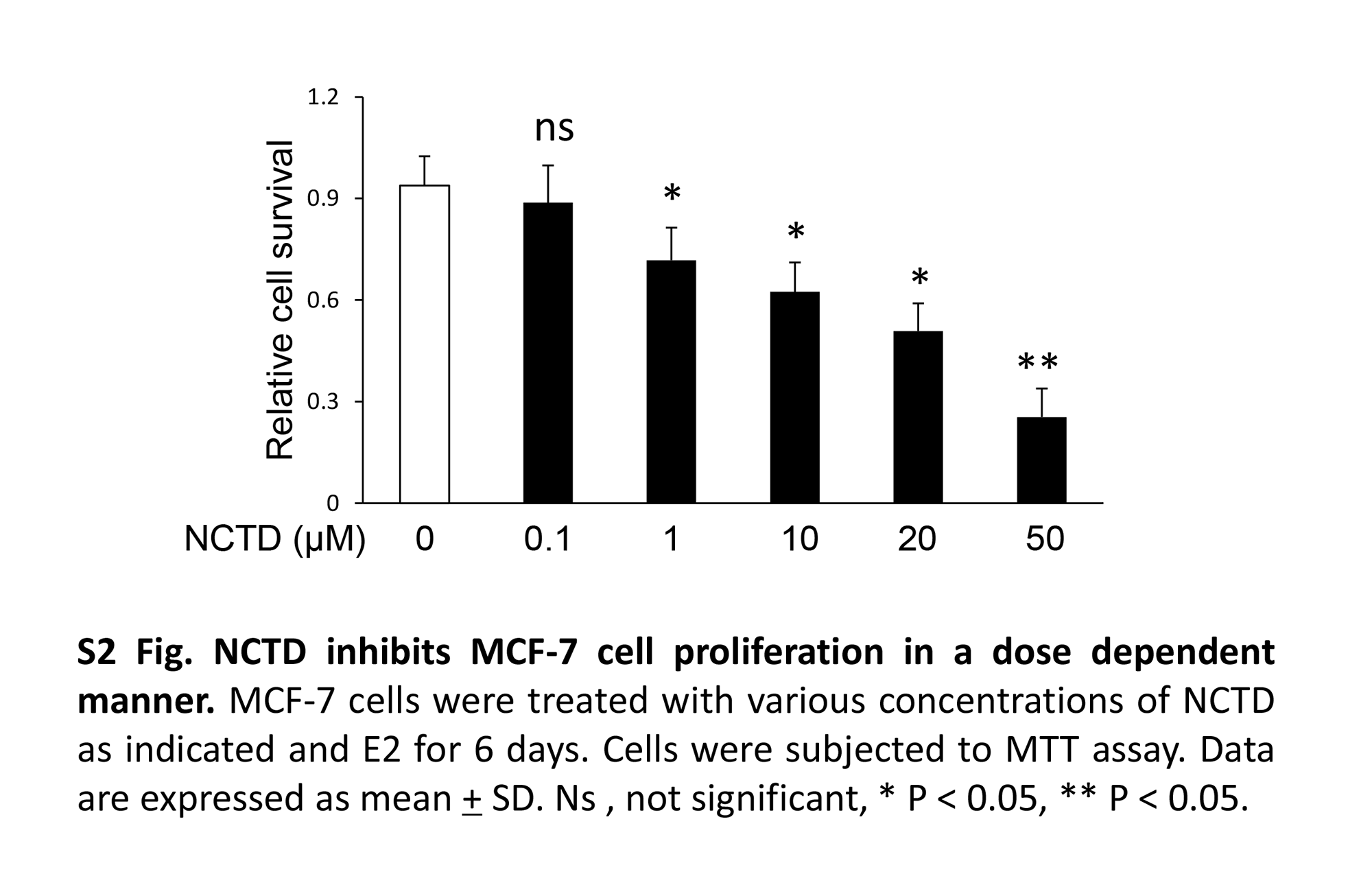

Supplement: S2 Fig — MCF-7 cells were treated with various concentrations of NCTD as indicated and E2 for 6 days. Cells were subjected to MTT assay. Data are expressed as mean ± SD. Ns, not significant, * P < 0.05, ** P < 0.05. (TIF) [file pone.0217181.s002.tif]

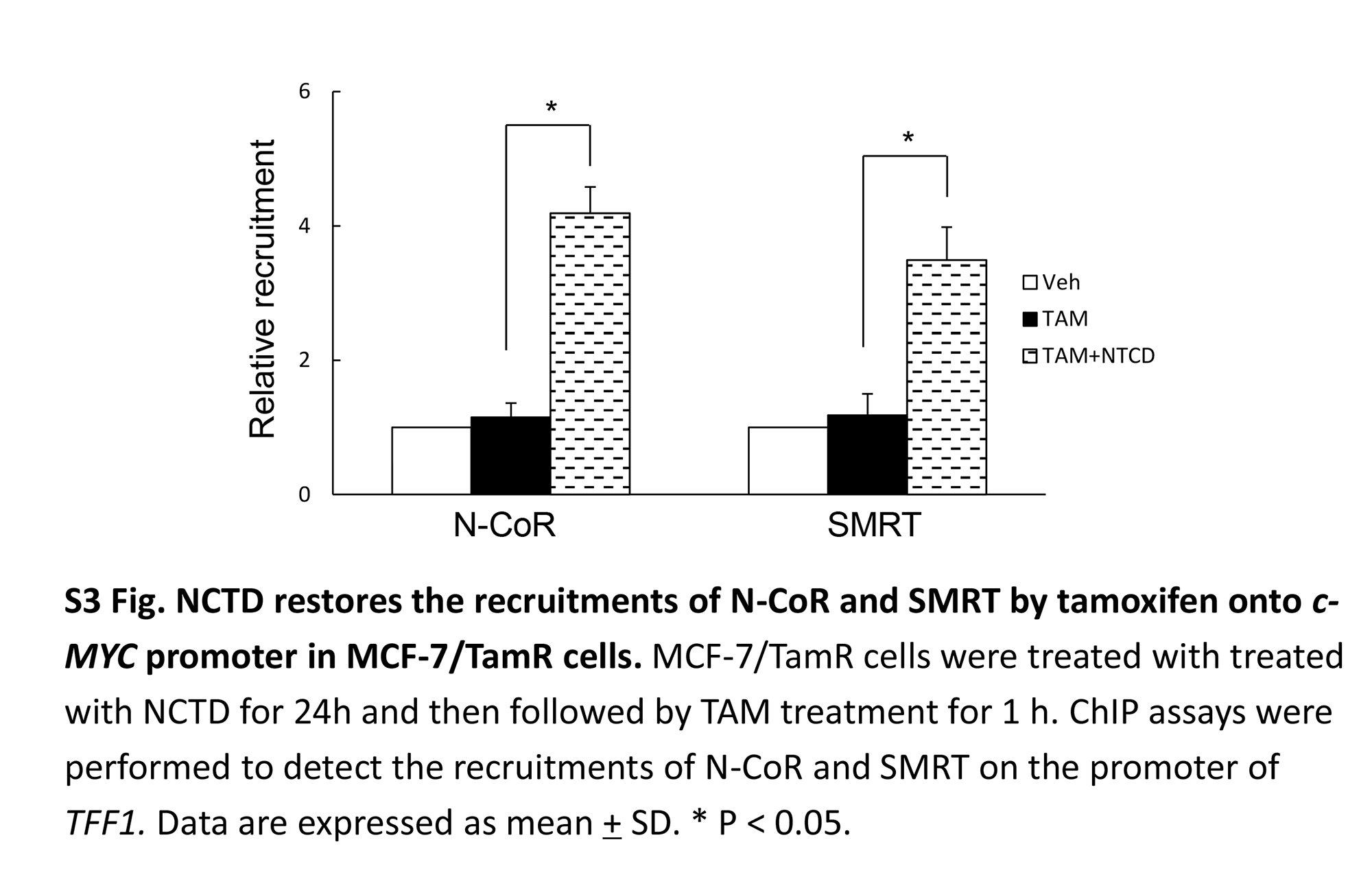

Supplement: S3 Fig — MCF-7/TamR cells were treated with treated with NCTD for 24h and then followed by TAM treatment for 1 h. ChIP assays were performed to detect the recruitments of N-CoR and SMRT on the promoter of TFF1. Data are expressed as mean ± SD. * P < 0.05. (TIF) [file pone.0217181.s003.tif]
